# Racial/ethnic minority and low-income hotspots and their geographic proximity to integrated care providers

**DOI:** 10.1186/1747-597X-8-34

**Published:** 2013-09-23

**Authors:** Erick G Guerrero, Dennis Kao

**Affiliations:** 1 School of Social Work, University of Southern California, 655 West 34th Street, Los Angeles, CA 90089, USA; 2 Graduate College of Social Work, University of Houston, Houston, TX, USA

**Keywords:** Integrated care, Low-income, Diverse communities, Geographic information systems

## Abstract

**Background:**

The high prevalence of mental health issues among clients attending substance abuse treatment (SAT) has pressured treatment providers to develop integrated substance abuse and mental health care. However, access to integrated care is limited to certain communities. Racial and ethnic minority and low-income communities may not have access to needed integrated care in large urban areas. Because the main principle of health care reform is to expand health insurance to low-income individuals to improve access to care and reduce health disparities among minorities, it is necessary to understand the extent to which integrated care is geographically accessible in minority and low-income communities.

**Methods:**

National Survey of Substance Abuse Treatment Services data from 2010 were used to examine geographic availability of facilities offering integration of mental health services in SAT programs in Los Angeles County, California. Using geographic information systems (GIS), service areas were constructed for each facility (*N* = 402 facilities; 104 offering integrated services) representing the surrounding area within a 10-minute drive. Spatial autocorrelation analyses were used to derive hot spots (or clusters) of census tracts with high concentrations of African American, Asian, Latino, and low-income households. Access to integrated care was reflected by the hot spot coverage of each facility, i.e., the proportion of its service area that overlapped with each type of hot spot.

**Results:**

GIS analysis suggested that ethnic and low-income communities have limited access to facilities offering integrated care; only one fourth of SAT providers offered integrated care. Regression analysis showed facilities whose service areas overlapped more with Latino hot spots were less likely to offer integrated care, as well as a potential interaction effect between Latino and high-poverty hot spots.

**Conclusion:**

Despite significant pressure to enhance access to integrated services, ethnic and racial minority communities are disadvantaged in terms of proximity to this type of care. These findings can inform health care policy to increase geographic access to integrated care for the increasing number of clients with public health insurance.

## Background

The substance abuse treatment (SAT) field in the United States faces an unprecedented challenge to reduce health disparities among racial and ethnic minority populations suffering from co-occurring substance abuse and mental health disorders [1-3]. Access to integrated care, referred here as provision of substance abuse and mental health treatment services is associated with improvement in process and health outcomes [4,5], making integration of co-occurring disorder treatment the most significant and cost-effective service delivery expansion in SAT [6,7]. However, the substance abuse and mental health treatment fields are characterized by different philosophies, approaches, and cultures that impede integration, coordination, or both of dual-diagnosis or co-occurring disorder treatment [8,9].

Health care reform in the United States, through the Affordable Care Act, will enable states and counties to provide funding and regulation for behavioral health organizations to further develop integrated care services and expand public insurance coverage for low-income and ethnic minority communities. Los Angeles County plans to expand eligibility for the public insurance program known as Medicaid, referred to as Medi-Cal in California, in 2014 for an estimated 1 million people, mainly Latino (40%) and African American (34%) residents [10]. SAT programs located in ethnic minority and low-income communities in L.A. County are poised to become primary intervention points for the diagnosis and treatment of co-occurring disorders. However, integrated care service providers may be limited in low-income and racial and ethnic minority communities in which they are highly needed. To examine the extent to which gaps exist in service coverage among minority and low-income communities, the current study geographically mapped the availability of integrated care among SAT programs in L.A. County and identified the probability of offering integrated care using these communities as main predictive factors.

### Co-occurring disorder treatment needs among low-income and minority groups

The literature on dual diagnosis has indicated that more than 40% of individuals with substance abuse issues also experience mental illness such as bipolar, depression, or anxiety disorders at some point during their lifetime [11,12]. In L.A. County, estimates have indicated that clients with dual disorders represent more than 44% of all individuals entering publicly funded treatment [4]. Seventy percent of these clients live in an urban area and have an ethnic minority background. In 2010, the treatment population in L.A. County was 43% Latino, 30% non-Latino White, 21% African American, and 4% Asian. Although L.A. County is one of the most populous and geographically expansive metropolises in the world, it contains discrete communities with large populations of African Americans, Asians, and Latinos [4].

Access to integrated mental health and substance abuse treatment is critical to achieve recovery for individuals suffering from co-occurring disorders. Yet, people face significant barriers to accessing this specialty treatment and the literature has suggested that members of racial and ethnic minority groups in particular are less likely to receive treatment due to lack of health insurance, lower socioeconomic status, and consistently high rates of unemployment [13]. In particular, studies have suggested that access to specialty mental health services in substance abuse treatment is limited among urban racial and ethnic minority communities [14,15].

### Geographic access to integrated care

Research on access to integrated mental health and substance abuse treatment has focused on the organization, management, and delivery of services. Yet, a growing concern related to access to integrated care for minority and low-income individuals is the burden of traveling to specialized providers. Geographic distance to care represents a significant source of health disparities for low-income individuals and minorities and although several factors contribute to access, high levels of residential segregation and imbalance of geographic proximity to needed services may negatively affect the health and well-being of disadvantaged communities [16].

Studies on health care service utilization have begun to discover place-based disparities involving a significant relationship between the racial and ethnic composition of communities and disparities in health care utilization or availability of physicians, mainly in African American and Latino communities [17]. These authors also posited that the racial and ethnic composition of neighborhoods affects the supply of health care providers [17]. Providers are discouraged to deliver services in low-income communities by low rates of reimbursement by public insurance or limited out-of-pocket self-pay revenue associated with high proportions of low-income and publicly insured African Americans and Latinos. Community-based SAT providers may be more prone than mainstream providers to establish their services in underserved areas. Yet, it may be the case that health care providers, including SAT providers, may avoid certain low-income and minority communities that are isolated from other specialty providers. By avoiding these areas with limited resources, SAT providers may affect access to care among minority clients in terms of longer wait times for treatment or more time spent traveling outside their low-resourced communities to obtain mental health care.

Growing evidence has suggested that significant disparities exist in access to mental health treatment providers among racial and ethnic minority groups and those living in high-poverty neighborhoods [18]. However, empirical evidence remains limited. Neighborhood poverty is considered a central factor in understanding and addressing racial and ethnic disparities in access to mental health care services. Impoverished communities affect the overall well-being of residents due to relatively higher rates of crime, public substance use, and homelessness in those communities [18]. Individuals suffering from mental health issues are also overrepresented in high-poverty neighborhoods. Federal and state agencies [19], as well as researchers and treatment providers, are concerned about the paucity of research regarding place-based disparities in access to care. Benefits from such research can inform evidence-based policy approaches to investing in community-based behavioral health care and promoting health equity [20].

Few if any studies regarding low income and racial/ethnic minority communities have focused on distance as a potential barrier to accessing specialty services, yet research has suggested that it is an important factor to promoting health equity by enhancing access to integrated care and improving completion rates [21-23]. Fortney et al. [22] studied 106 clients receiving treatment for depression and found that increased travel time to providers was significantly associated with fewer visits. Increased travel time was also associated with a greater likelihood of receiving less effective care [22]. Similarly, Beardsley et al. [21] focused on the distance traveled by 1,735 clients to various outpatients SAT programs in an urban setting. They found that distance was strongly correlated with treatment completion and higher retention rates; specifically, clients who traveled less than 1 mile were more likely to complete treatment than those who traveled farther. Transportation is one of the most noted barriers to treatment for Latinos and other low-income minorities because these populations are more likely to lack driver’s licenses and auto insurance, have poor public transportation options, and may need to travel significant distances to engage in treatment that meets their cultural and health care service needs [24].

Various studies have employed GIS to examine the distribution of treatment centers and the relationship between distance and access to SAT [25] or Spanish-language services [26]. For instance, using 2010 U.S. Census data on Latino communities in L.A. County and 2010 national data from the Substance Abuse and Mental Health Services Administration (SAMHSA) on outpatient treatment facilities offering services in Spanish, Guerrero et al. [24] found travel distances from neighborhoods heavily populated by Latinos to Spanish-language services that ranged from 4 to 6 miles. The use of GIS to examine access to needed services has become a critical exploratory approach to support evidence-based policy that seeks to maximize public resources and improve population health. Although this study was exploratory in nature, based on the existing research literature we expected that low-income and ethnic minority communities would have fewer facilities that offered integrated care.

## Methods

### Sampling frame

We relied on publicly available data collected in 2010 from the National Survey on Substance Abuse Treatment Services (NSSAT-S). The source was the directory of SAT facilities drawn from NSSAT-S and publicly available through SAMHSA’s Behavioral Health Treatment Services Locator (http://findtreatment.samhsa.gov). These data include the location and services provided by treatment providers and were created to serve as a treatment referral resource for the general public. We used the search tool to identify and download a data table of treatment facilities located in Los Angeles County, California.

It is important to note that by using this source of data, we did not compromise the deidentified nature of these data; we did not use program names and only relied on aggregate data to present results. Moreover, the maps presented in this study were drawn at a scale that would make it difficult to identify any specific program. The N-SSATS data come from SAMHSA’s annual census of drug treatment facilities. Although the survey is conducted annually to collect cross-sectional data, only N-SSATS data collected in 2010 were used for this study because census data on low-income and minority communities were collected in 2010 as well. More information about the sampling frame of N-SSATS is available from SAMHSA [27].

In the N-SSATS data, a facility is defined as the point of delivery of substance abuse treatment services (i.e., physical location). Although the dataset included facilities operated by federal agencies, including the Department of Veterans Affairs, the Department of Defense, and the Indian Health Service, facilities connected to the military and criminal justice systems were excluded. This was mainly because military and criminal justice facilities have different operational and service delivery structures and intake criteria, making them inappropriate to compare with regular programs in terms of access by community residents.

N-SSATS data are particularly appropriate for analysis of treatment service trends and comparative analyses for the nation, regions, states, and counties. But N-SSATS does not provide geographic information below the county or Metropolitan Statistical Area level in public use files to protect the identity of providers. Also, certain data limitations must be taken into account when interpreting data from the N-SSATS; for instance, the survey is voluntary and there is no adjustment for nonresponse (approximately 4%). Further, the N-SSATS is a point-prevalence survey that provides a cross-sectional snapshot of yearly statistics.

To identify racial/ethnic minority and low-income clusters, or *hot spots*, in L.A. County, racial/ethnic data were drawn from the 2010 Census and poverty data were based on 2006–2010 American Community Survey data. These two sources of data have been used in other GIS studies to examine hot spots in the Los Angeles County area [24,26].

### Selection procedure

To systematically identify and compare SAT facilities with similar organizational structures, three selection criteria were used when searching the SAMHSA database. A facility was included if it was located in L.A. County and (1) was primarily a substance abuse treatment facility (excluding all medical care or facilities that primarily provide mental health services); (2) provided mainly outpatient services; and (3) was not part of a solo practice or connected to the military or criminal justice systems.

### Measures

#### Outcome measure: facilities offering substance abuse and mental health services

Our single outcome focused on SAT facilities that offered integrated care. The survey item asked respondents about the main focus of their services. We selected the variable “FOCUS 3,” which in N-SSATS represents SAT facilities that provide “mix of mental health and substance use” services. We determined the likelihood of a SAT facility offering integrated care, using each facility’s coverage of racial/ethnic minority and low-income hot spots as the main predictors.

#### Independent variables: facility coverage of racial/ethnic minority and low-income hot spots

Our main independent variables were each facility’s coverage of hot spots with high concentrations of (1) African Americans, (2) Asians, (3) Latinos, and (4) low-income households. For each facility, service areas were computed to represent the surrounding area within a 10-minute drive to the facility (using any combination of roads). The 10-minute threshold was drawn from the health services research literature, as well as the literature focused on food deserts [28,29]. The geographic coordinates of each facility were geocoded based on its address. Using a street network based on Esri’s StreetMap data [30], the “service area” tool in ArcGIS was used to generate boundaries around each facility.

To obtain this hot spot coverage measure, we needed to identify racial/ethnic minority and low-income hot spots as an intermediary step. We used spatial autocorrelation analysis to identify statistically significant clusters of census tracts with large concentrations of African Americans, Asians, Latinos, and low-income households (defined as below the federal poverty level). Spatial autocorrelation refers to the interdependence or interrelatedness among geographic units (in this case, census tracts), particularly units that are closer to one another [31,32]. Spatial autocorrelation analysis, therefore, statistically compares neighboring geographic units with similarly high or low values of a particular characteristic and identifies hot spots or clusters of similar geographic units. Based on Anselin’s work on local indicators of spatial association [32], we calculated local Moran’s I values to determine the presence of significant clustering of census tracts based the proportion of African Americans, Asians, Latinos, and low-income individuals (as a percentage of the total population). We used a queen-based contiguity weight matrix, which considers neighboring census tracts as having either common boundaries or vertices. Separate analyses were conducted for each demographic characteristic (i.e., African American, Asian, Latino, and low-income households), resulting in individual hot spot map layers. For this study, we included clusters of census tracts with high proportions of each group.

### Analytic framework

Data from the 2010 Census and SAMHSA’s facility locator were managed and analyzed using ArcGIS 10.0, a mapping software system designed to facilitate the collection, management, and analysis of spatially referenced information and associated attribute data [33]. A three-phase spatial analytic approach was subsequently used to measure the potential accessibility of integrated care facilities for minority and low-income communities. Data management and manipulation, geocoding, and map production were conducted in ArcGIS 10 [33] and the network (service area) analysis was conducted using Esri’s Network Analyst extension [34]. The spatial autocorrelation analysis was conducted with OpenGeoDa 0.9.9.10 [35,36].

### Statistical analysis

Data analysis was performed using STATA/SE (version 12) for all procedures. We conducted independent sample *t* tests to determine differences between hot spot coverage of facilities with and without integrated care. In addition, we employed logistic regression models with robust standard error specification for the dichotomous outcome (0 = *facility does not offer co-occurring disorder treatment*, 1 = *facility offers co-occurring disorder treatment*). Goodness-of-fit tests were used to analyze the appropriateness of the regression model.

The statistical analysis complemented the GIS analysis and consisted of two steps. In the first step, four univariate logistic regression models were used to examine the bivariate relationship between a facility’s hot spot coverage (for each of the four different groups) and its odds of offering integrated care. We started with the most populous group in Los Angeles County (Latinos; Model 1), followed by African Americans (Model 2), Asians (Model 3), and low-income households (Model 4). The second step relied on three multivariate logistic regression analyses with robust standard errors using a cumulative approach. Because the conceptual framework indicated that communities in Los Angeles County are diverse and feature high rates of low-income individuals, particularly in African American and Latino neighborhoods, a hierarchical nested regressions analysis was conducted to capture the unique explained variance in the outcome for each hot spot across three cumulative statistical models (Models 5–7). Examination of Wald Chi-square tests and quadrature statistics were examined as a goodness-of-fit test to determine the appropriateness of our logistic regression models.

## Results

### GIS mapping of hot spots

**Figure 1:**
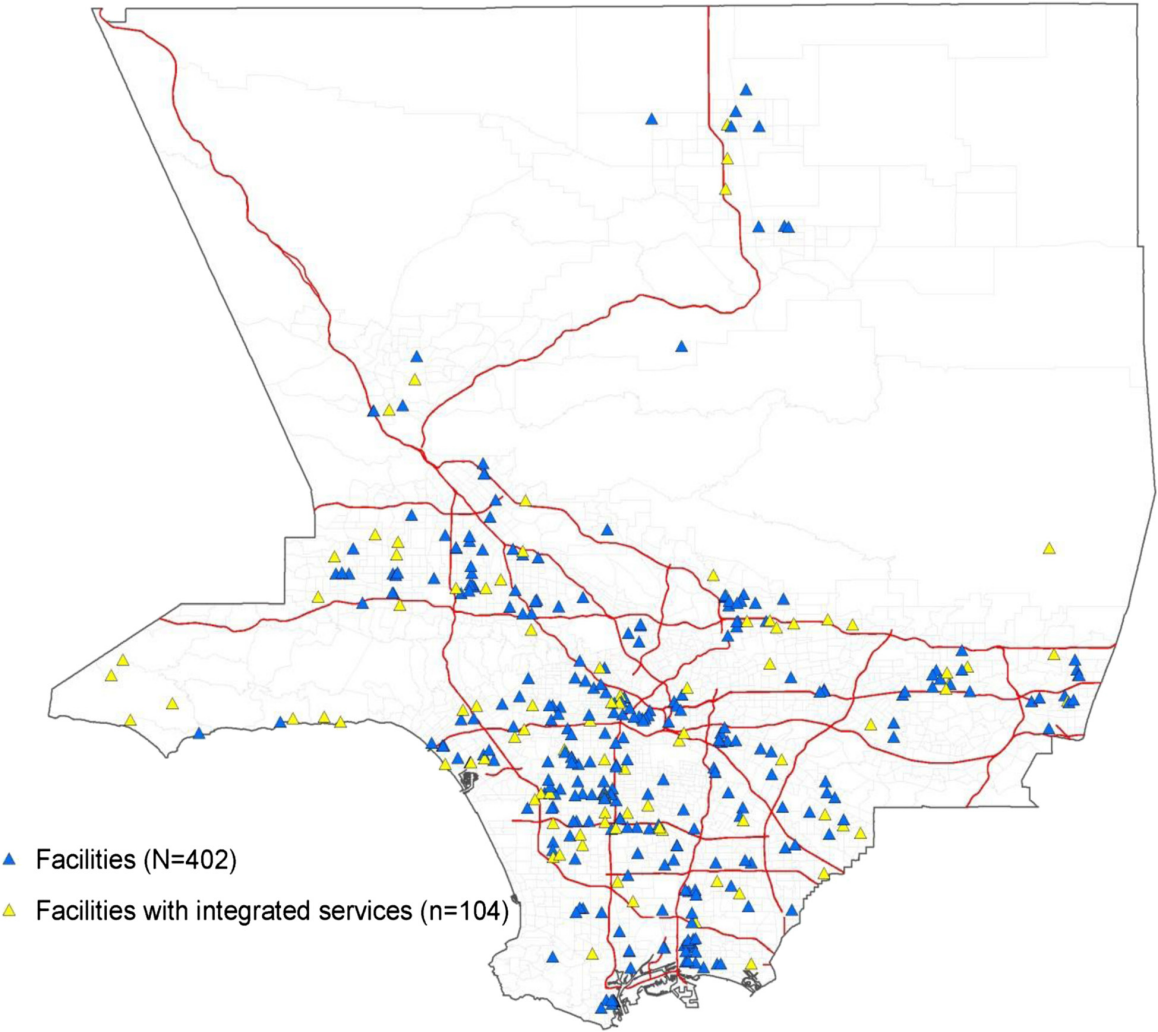
Map of SAT facilities and integrated care services, Los Angeles County, California.

**Figure 2:**
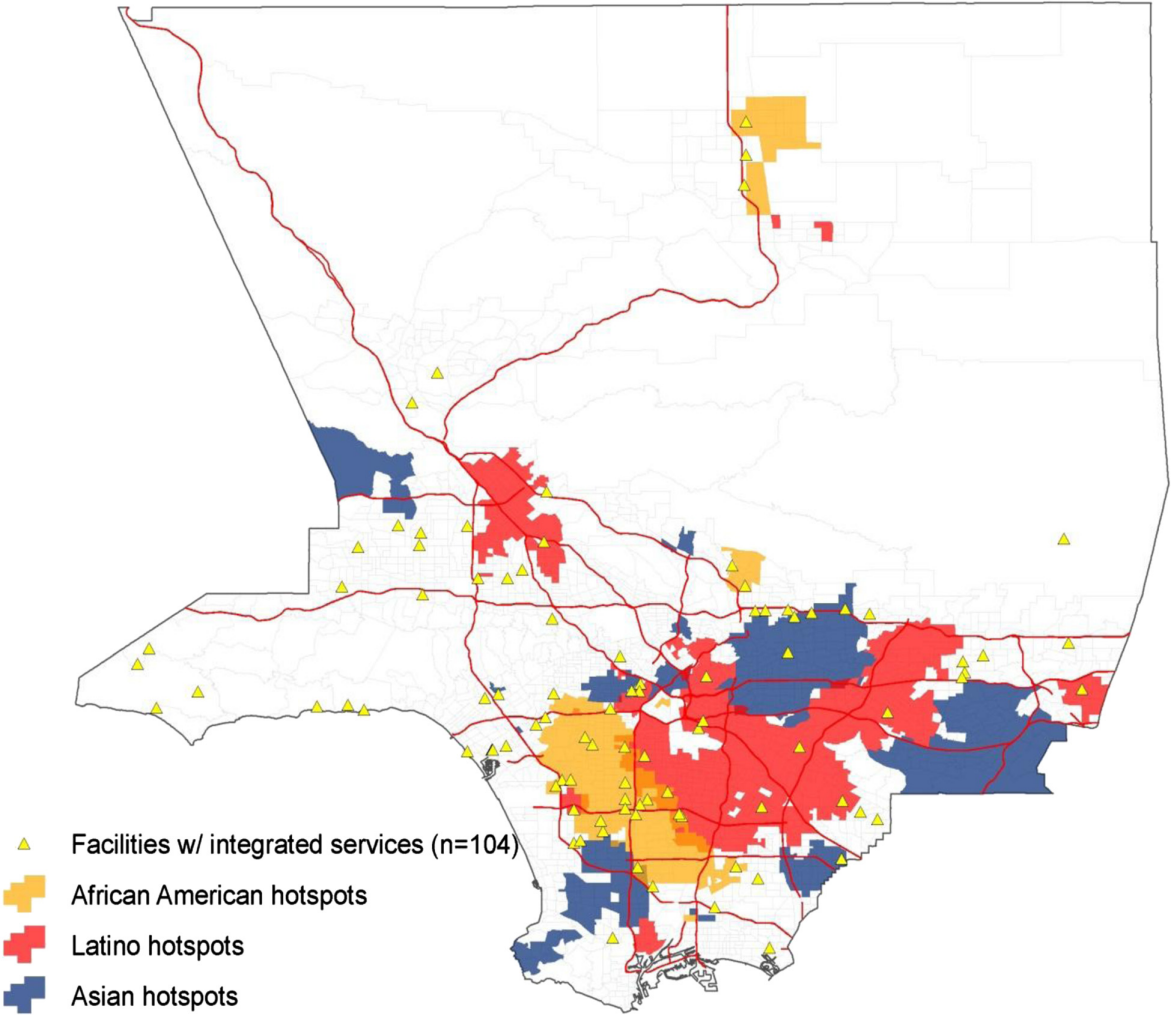
Map of hotspots by ethnic group and SAT facilities offering integrated care services.

**Figure 3:**
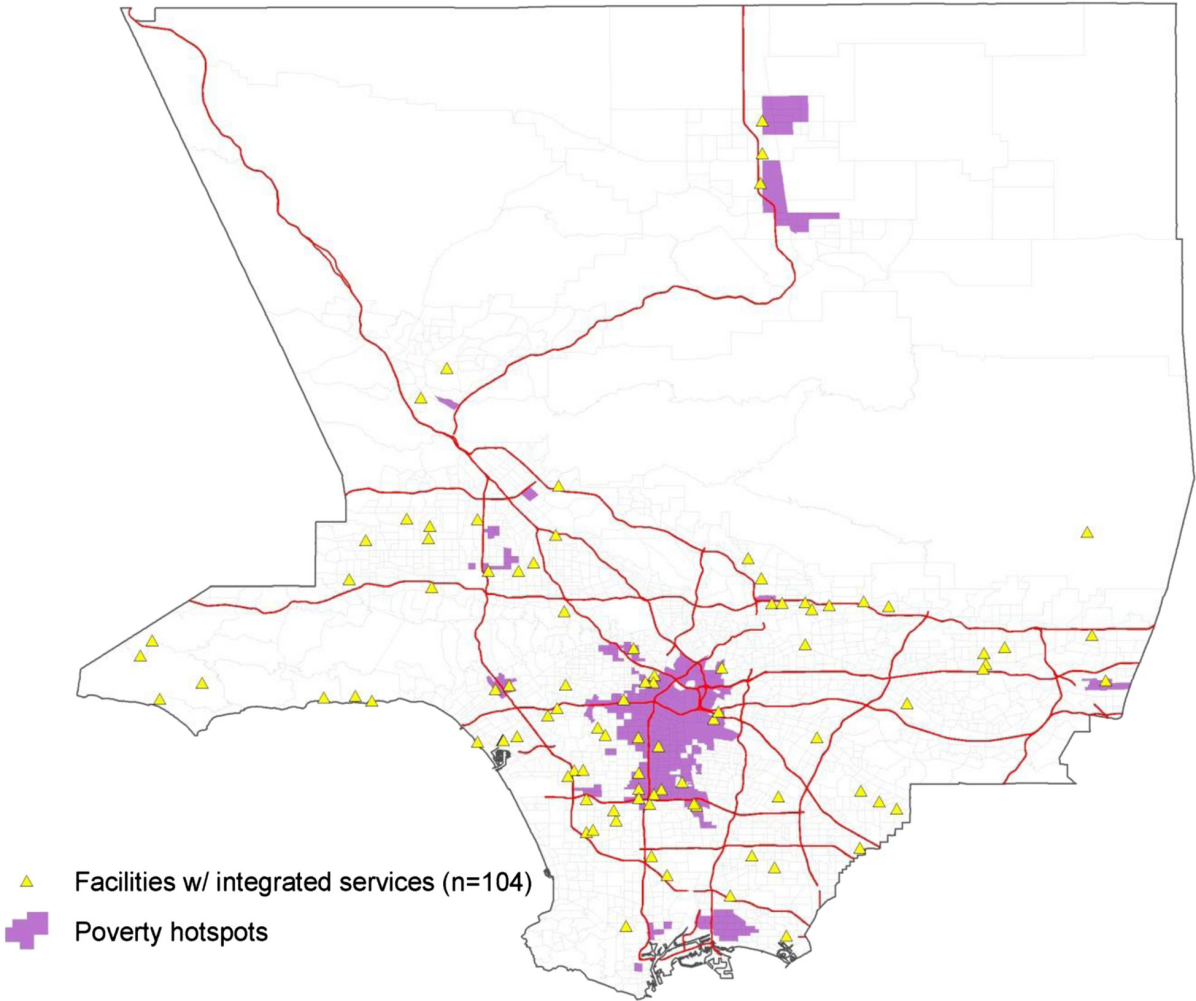
Map of poverty hotspots and SAT facilities offering integrated care services.

Figure 1 shows the geographic distribution of facilities that treat both substance abuse and mental health disorders (104 of 402 SAT facilities). Although the geographic distribution of integrated care facilities appears evenly spread throughout Los Angeles County, the map in Figure 2 shows specific hot spots of African American, Asian, and Latino communities with limited access. Based on the maps, the presence of facilities offering integrated care seemed to be particularly lacking in Latino and Asian communities. Only 20 out of the 100 facilities (20.0%) in Latino communities and only 5 out of the 24 facilities (20.8%) in Asian communities provided integrated care. In contrast, 25 out of the 87 facilities in the African American hot spots (28.7%) provided integrated care. Still, in all of these racial/ethnic minority communities, substance abuse and mental health services were integrated in less than a third of the facilities. In addition, Figure 3 shows the areas in which low-income households were prevalent; only 25 of the 91 facilities (27.5%) in those hot spots offered integrated care.

### The relationship between hot spots and provision of integrated care

Among facilities providing integrated care, the hot spot coverage was 17.8% for African Americans, 17.6% for Latinos, and 10.2% for Asians (results only shown in text). Based on these results, Asian communities seemed to have the least access to facilities providing integrated care. However, based on independent sample *t* tests, we found that the difference between hot spot coverage of facilities with and without integrated care was only significant for Latino hot spots. Specifically, facilities with integrated care covered significantly less of the Latino hot spots compared to facilities without integrated care (17.6% vs. 23.2%, respectively; *t* = 2.26, *p* < .01). For the other two ethnic minority groups, the difference in hot spot coverage between facilities with and without integrated care was negligible: 17.8% versus 17.0% for African American hot spots (*t* = −0.31, *p* > .05) and 10.2% versus 9.7% for Asian hot spots (*t* = −0.36, *p* > .05).

Logistic regression results for facility coverage of ethnic and low-income hot spots and the likelihood of offering integrated care showed that facilities with greater Latino hot spot coverage were less likely to offer integrated care (Table 1, Model 1). Facility coverage of the other hot spots—African American (Model 2), Asian (Model 3) and low-income households (Model 4)—were not statistically significant.

**Table 1:** Bivariate logistic regressions on SAT facility racial/ethnic and poverty hot spot coverage and the odds of providing integrated care (n = 402 facilities)

	**Model 1**		**Model 2**		**Model 3**		**Model 4**	
**Facility hot spot coverage**	***OR***	**95% CI**	***OR***	**95% CI**	***OR***	**95% CI**	***OR***	**95% CI**
Latino	0.28*	0.09-0.85						
African American			1.17	0.44-3.14				
Asian					1.17	0.44-3.14		
Low-income							0.29	0.70-1.21
*R*^2^	.012		.0003		.0002		.007	
Log likelihood	−227.16		−229.76		−229.77		−228.29	
Wald χ^2^ (*df*)	5.32 (1)		0.13 (1)		0.10 (1)		3.06 (1)	

*p < .05; *OR* odds ratio, *CI* confidence interval, *df* degrees of freedom, hot spot coverage defined as the proportion of a facility’s service area that overlaps each hot spot.

In the cumulative regression models, the negative effects of Latino hot spot coverage were consistently statistically significant after adding hot spot coverage for African Americans and Asians (Table 2, Models 5 and 6). However, when including facility coverage of low-income hot spots in Model 7, the relationship between coverage of Latino hot spots and provision of integrated care was no longer significant.

**Table 2:** Multivariate logistic regression on SAT facility racial/ethnic and poverty hot spot coverage and the odds of providing integrated care (n = 402 facilities)

	**Model 5**		**Model 6**		**Model 7**	
**Facility hot spot coverage**	***OR***	**95% CI**	***OR***	**95% CI**	***OR***	**95% CI**
Latino	0.28*	0.09-0.86	.026*	0.08-0.82	0.38	0.12-1.26
Asian	1.36	0.25-7.43	1.48	0.26-8.24	1.44	0.26-7.90
African American			1.44	0.52-4.01	2.61	0.74-9.17
Low-income					0.21	0.03-1.47
*R*^2^	.012		.013		.019	
Log likelihood	-.227.10		−226.86		−225.53	
Wald χ^2^ (*df*)	5.44 (2)		5.93 (3)		8.58 (4)	

*p < .05; *OR* odds ratio, *CI* confidence interval, *df* degrees of freedom, hot spot coverage defined as the proportion of a facility’s service area that overlaps each hot spot.

To identify this potential interaction, we tested the interaction of Latino and poverty hot spot coverage and added it to Model 7 (results only shown in text). This interaction was statistically significant and associated with a large odds ratio (*OR* = 1.70 × 10^6^, *p* < .01, Wald χ^2^ = 20.2, *df* = 5).

## Discussion

In this study, we addressed questions about access to facilities offering integrated substance abuse and mental health disorder treatment among ethnic minority and low-income communities. Geographic availability of integrated care providers was limited in racial/ethnic minority and low-income communities, supporting findings from other studies on disparities in access to health care [17,18]. In particular, findings in this study suggest that the geographic distance between Latino hot spots and the closest facility offering integrated care is significant enough to be considered an access barrier. Although Latinos represent the most populous group in L.A. County, some Latino communities are located in isolated and underserved regions and report some of the highest rates of low-income households.

Integrating mental health into SAT programs is challenging in low-resourced communities. This challenge is further compounded by the lack of financial and policy integration in L.A. County. In this county, departments of mental health and substance abuse treatment are organized as separate systems, making it difficult to design and fund integrated behavioral health services. Because the main principle of health care reform is enhancing access to health care for low-income individuals and promoting health equities, both departments in L.A. County are currently developing initiatives to diversify the skills of their workforce and train staff members in co-occurring behavioral health issues [37]. However, these efforts should also include funding provision of specialty care in under resourced low-income and minority communities. Consistent with other initiatives that seek to stimulate business and service activities in low-income communities, the county can create incentives for public and private SAT organizations to offer specialty care services in underserved areas.

### Data limitations

Data-related limitations should be considered when interpreting our findings. Due to the nature of the existing data, the current analysis considered ethnic minority and low-income individuals’ place of residence and did not account for “daily activity spaces” [38]. For example, individuals may be more concerned about travel distance from their place of employment as opposed to their residence. Also, to generate each facility’s service area, this analysis only considered travel time to each facility by car, which is a function of the network of freeways and roads used to travel to any given facility and official speed limits. However, there are other factors that may influence access, such as the availability of transportation (private or public), peak travel hours, and traffic conditions. Los Angeles County is known for its car culture and insufficient public transportation; hence, our study focused on individuals traveling to a facility from their residential community via automobile. As noted above, we decided on a 10-minute driving radius based on our review of the literature, however, we acknowledge that these results may vary depending on other thresholds. Future research should note that driving thresholds may vary depending on the study site, but sensitivity analyses of different thresholds may help to decipher these issues. It is also important to note that due to the inherent nature of spatial data, this type of analysis is sensitive to the effects of scale and aggregation [31]. The boundaries of census tracts—used as a proxy for racial/ethnic communities—are designated arbitrarily by the U.S. Census Bureau and do not depict particular communities. Yet, the use of census tract data is common in this type of analysis to describe demographic density [39].

Additionally, we acknowledge that the geographic areas presented in this study may have access to integrated care through other providers (primary medical and mental health settings). Although those providers are also scarce in minority and low income communities [17], together with SAT they may increase access to specialty care.

Finally, although we used the same N-SSATS survey data collected in 2010 for the GIS and statistical regression analyses, different versions of N-SSATS data were used. The statistical analysis of N-SSATS used data on organizational and service characteristics, which did not have identifiers, whereas the GIS analysis relied on SAMHSA facility locator N-SSATS data with location and identifiers but limited information on organizational characteristics. However, because the N-SSATS data on treatment facilities represent all treatment providers in the country, both samples were highly correlated. Despite these challenges, the data and methods used here allowed us to develop preliminary evidence of travel burden in distinct areas of L.A. County.

## Conclusions and implications

Findings from this study provide valuable insight about the access of racial/ethnic communities to integrated care. Although Asian communities had the least coverage in terms of number of facilities providing integrated care, Latino communities were significantly less likely to be located near facilities that provided integrated care than those without integrated care. Latino communities also represented some of the most impoverished areas in the county. Hence, both Latino and low-income communities had limited access to integrated care. Finally, although we did not include Whites in our analysis, the lack of integrated care in low-income communities suggests that this issue may also affect low-income Whites who may also face geographic barriers to services.

In sum, access to care in terms of geographic proximity of providers to ethnic and low-income communities varies based on ethnic group, but was most limited among Latino and low-income communities. Although driving time may vary in different cities, the findings still suggest that that certain communities have less access to integrated care than other communities—particularly those that are more reliant on public transportation. These place-based disparities involving a significant relationship between the racial and ethnic composition of communities and disparities in health care utilization or availability of providers [17] may be reduced with the effective implementation of health care reform. Through the expansion of public insurance coverage, this legislation seeks to enhance population health in underserved and low-income communities by increasing access to integrated care [20]. Study findings have implications for the implementation of expanded public insurance coverage. State and local governments may be able to rely on public insurance reimbursement rates and other public funding mechanisms to incentivize health care providers to serve the low-income and minority communities identified in this study, thus decreasing disparities in access to integrated care.

## Competing interests

The authors declare that they have no competing interests.

## Authors’ contributions

EG participated in the design of the study, developed the first draft of the literature review, and performed the statistical analysis. DK participated in the design and coordination of the study, conducted the GIS analysis, and helped to draft the final manuscript. Both authors read and approved the final manuscript.

## Authors’ information

EG is an assistant professor at the University of Southern California, School of Social Work. His funded research focuses on the geographical and organizational context of Latino health disparities, the implementation of culturally responsive and evidence-based practices, and the integration of behavioral health and primary care. DK is an assistant professor at the University of Houston, Graduate College of Social Work. His funded research focuses on access to behavioral health services and the use of geographic information system data to inform policies on service delivery.
